# High-efficiency robust perovskite solar cells on ultrathin flexible substrates

**DOI:** 10.1038/ncomms10214

**Published:** 2016-01-11

**Authors:** Yaowen Li, Lei Meng, Yang (Michael) Yang, Guiying Xu, Ziruo Hong, Qi Chen, Jingbi You, Gang Li, Yang Yang, Yongfang Li

**Affiliations:** 1 Department of Materials Science and Engineering, University of California, Los Angeles, California 90095, USA; 2 Laboratory of Advanced Optoelectronic Materials, College of Chemistry, Chemical Engineering and Materials Science, Soochow University, Suzhou 215123, China

## Abstract

Wide applications of personal consumer electronics have triggered tremendous need for portable power sources featuring light-weight and mechanical flexibility. Perovskite solar cells offer a compelling combination of low-cost and high device performance. Here we demonstrate high-performance planar heterojunction perovskite solar cells constructed on highly flexible and ultrathin silver-mesh/conducting polymer substrates. The device performance is comparable to that of their counterparts on rigid glass/indium tin oxide substrates, reaching a power conversion efficiency of 14.0%, while the specific power (the ratio of power to device weight) reaches 1.96 kW kg^−1^, given the fact that the device is constructed on a 57-μm-thick polyethylene terephthalate based substrate. The flexible device also demonstrates excellent robustness against mechanical deformation, retaining >95% of its original efficiency after 5,000 times fully bending. Our results confirmed that perovskite thin films are fully compatible with our flexible substrates, and are thus promising for future applications in flexible and bendable solar cells.

## Abstract (web summary)

Most efficiency values of flexible devices lag behind those on rigid substrates. Here, Li *et al*. fabricate a flexible perovskite solar cell on a silver-mesh/conducting polymer and demonstrate a power conversion efficiency of 14% and greater than 95% of its original efficiency after 5,000 times bending.

## Graphical abstract

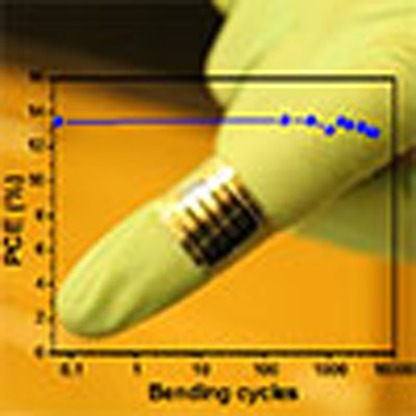

Solution processing perovskite-based photovoltaic cells have reached a power conversion efficiency (PCE) of 20% (ref. 1). The rapid progress in both device efficiency^2^,^3^,^4^,^5^,^6^,^7^,^8^ and stability^9^,^10^,^11^ indicates the potential application of perovskite materials in next generation solar cells. There are two device architectures currently dominating in the field of perovskite solar cells (pero-SCs): mesoporous type and planar heterojunction. Solar cells using a TiO_2_ mesoporous layer as a scaffold still lead in terms of efficiency. In addition to using a thick perovskite layer for stronger light absorption, obtaining high-quality TiO_2_ nanocrystals and forming high-quality interfacial contacts with the perovskite layer greatly improves device performance by reducing interface recombination. The TiO_2_ scaffold layer usually requires high temperature annealing at 450 °C that is commonly used in pero-SCs^12^,^13^.

As an alternative to the mesoporous pero-SCs, the planar heterojunction design, which features a straightforward fabrication procedure, is also becoming attractive^5^,^14^,^15^. In this device architecture, organic semiconductor hole- and electron-transporting layers, which have also been widely investigated, are proper candidates for high-throughput low temperature manufacturing (<150 °C), opening the possibility of constructing flexible photovoltaic cells on various polymer substrates. Most of the flexible pero-SCs have been reported with PCEs in the range of 6–10% (refs 16, 17, 18, 19). Jung and colleagues have reported a perovskite device on polyethylene naphthalate (PEN)/indium-tin-oxide (ITO) based flexible substrates, giving a 12.2% PCE. 50% loss in initial PCE was observed after 1,000 fully bending cycles^20^. More recently, Seok and colleagues reported a flexible pero-SCs with a PCE of 14.85% based on the similar substrate^21^. It is catching up with the state-of-the-art for flexible thin film solar cells, such as CIGS, CdTe and so on^22^,^23^.

Still most efficiency values of flexible devices lag behind those of the references on rigid substrates, and the bending durability of PEN/ITO is subpar. The major deficiencies arise from the relatively low conductivity and poor mechanical robustness of the transparent electrode on polymer substrates. Transparent conductive oxide (TCOs) electrodes, such as ITO and Al-doped ZnO usually have sheet resistance ∼10 ohm sq^−1^, increasing series resistance of the photovoltaic cells. Besides, TCOs are normally fragile against repeated bending, which has been recognized as the origin of device degradation/failure^20^,^24^. Going beyond flexible solar cells, wearable electronics require high durability against bending. It imposes further challenges to build up highly flexible pero-SCs. Until recently, the reported mechanical bending test of pero-SCs ended up with the failure of TCO electrodes, and whether perovskite thin films can endure multiple-times bending still remains unknown. Consequently, TCO-free electrodes or even indium-free electrodes are desirable owing to the poor mechanical robustness of poly-crystalline metal oxide films and the scarcity of indium and so on. Thin film conductors based on carbon materials^25^ and metal nanowires^26^ have been successfully demonstrated in flexible pero-SCs. However, the unsatisfying PCEs still limit their further applications, which is possibly ascribed to the imperfect perovskite film or poor contact between the neighbouring layers including the electrode, interfacial layer and perovskite film. The substrate of the flexible electrode is particularly important as well. Although the planar heterojunction pero-SC can effectively lower the annealing temperature below 150 °C, it is still higher than the glass-transition temperature of typical flexible substrates such as polyethylene terephthalate (PET) and PEN. As a result, it is desirable to develop flexible substrates or technology which are able to stand an annealing temperature of 150 °C. This is also critical for the roll-to-roll manufacturing of high-performance flexible pero-SCs. Therefore, for the flexible pero-SCs, the flexible electrodes and substrates should have sufficient thermal stability, high electrical conductivity and optical transmission, bending durability, facilitating growth of high-quality perovskite crystalline films.

Recently, a flexible PET substrate with embedded Ag-mesh (FEAM) was successfully fabricated via nano-imprinting technique, and used as electrodes in flexible optoelectronic devices^27^,^28^,^29^. In combination with high-conductivity transparent conducting polymer (Clevios PH1000), the hybrid electrode with high lateral conductivity can be readily fabricated for wide application in thin film devices. Our previous work shows that this hybrid electrode substrate with thickness of 200 μm exhibits a sheet resistance as low as 3 ohm sq^−1^ (ref. 28) The promising advantages of this hybrid electrode such as high flexibility and robustness, low sheet resistance and high transmission (78%) strongly suggest its future applications in flexible solar cells.

In this work, we integrate FEAM/PH1000 hybrid electrode into perovskite-based photovoltaic cells, and demonstrate an ultrathin flexible device delivering a PCE of 14.0%. This value is the highest among those pero-SCs based on TCO-free pero-SCs, and more importantly the device exhibits superior durability against mechanical bending over 5,000 times with 5-mm curvature and extremely high bending stability through rational device design. To fabricate the FEAM, we adopt low coverage, hexagonal Ag-mesh embedded in the ultrathin (∼57 μm) ultraviolet-resin-coated PET substrate that ensures high optical transmission. The bottom of the ultrathin FEAM substrate is laminated on a 100-μm-thick highly hardened PET protection film for thermal processing. It can be readily peeled off after completing the device, giving the ultrathin flexible pero-SCs.

## Results

### The structure and properties of FEAM-based hybrid electrode

**Figure 1:**
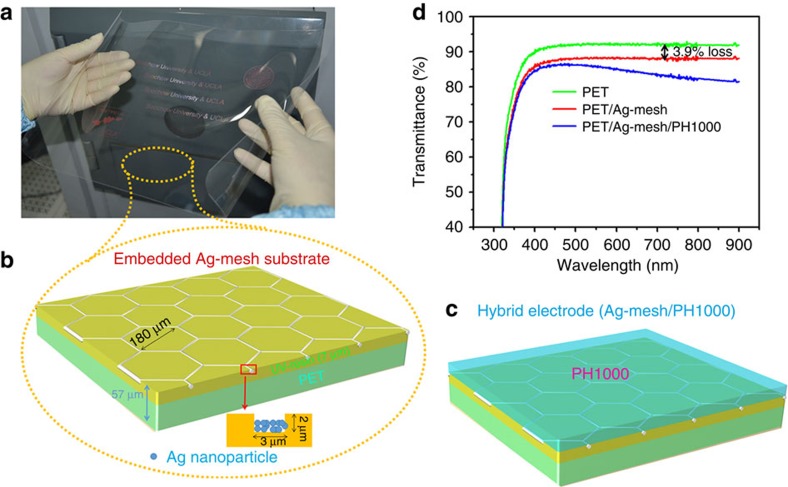
Schematic illustration of the FEAMs substrate and hybrid electrode. (**a**) An image of the large-area FEAMs substrate. (**b**) The structure of the FEAMs substrate with detail parameters. (**c**) The diagram for the hybrid electrode (PET/Ag-mesh/PH1000). (**d**) Transmission spectra of bare PET, PET/Ag-mesh, PET/Ag-mesh/PH1000-based substrates.

Ultrathin FEAMs substrate can be readily scaled up via roll-to-roll nano-imprinting, which makes testing the feasibility of this type of substrate for applications in pero-SCs the main motivation of this work. Figure 1a shows a 15-inch FEAM sheet exhibiting excellent flexibility and high transmission in the visible region. The substrate structure is shown in Fig. 1b. The substrate consists of periodically hexagonal Ag-mesh (diagonal length of 180 μm, width of 3 μm and height of 2 μm) embedded in ultraviolet-resin coated on PET, giving a substrate with total thickness of 57 μm. It is noteworthy that the coverage rate of Ag-mesh is 3.2%, which causes very low optical loss of <4% according to Fig. 1d. As the Ag-mesh period is several orders of magnitude larger than the visible wavelength, there is no significant light diffraction and scattering^29^. The hybrid electrode substrate PET/Ag-mesh/PH1000 was formed by spin-coating PH1000 onto PET/Ag-mesh substrate, then annealing at 120 °C for 20 min (Fig. 1c). The 150-nm-thick PH1000 layer shows smooth and continuous coverage on the PET surface with a root mean square (RMS) roughness of 2.0 nm (Supplementary Fig. 1a). According to Fig. 1d, the resulting hybrid PET/Ag-mesh/PH1000 electrode shows a transmission of 82–86% in the visible region, with an excellent sheet resistance of ∼3 ohm sq^−1^, almost one-third lower than that of commercial ITO-glass substrates. It should be noted here that the groove is not completely filled with Ag nanoparticles during the fabrication, resulting in an ∼500-nm-height difference between the Ag-mesh and the substrate surface. However, PH1000 still forms sufficient contact with the Ag-mesh as discussed later.

### FEAM substrate-based flexible pero-SCs design

**Figure 2:**
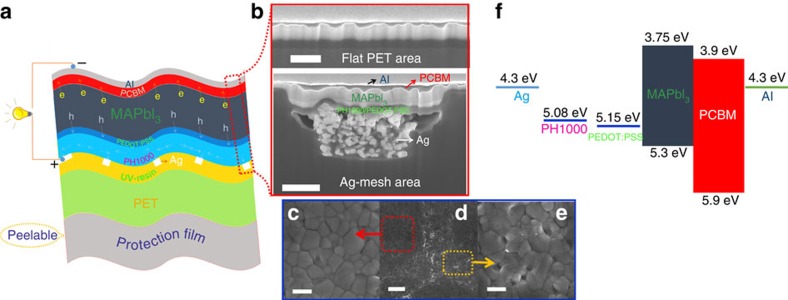
Flexible pero-SCs architecture and morphology. (**a**) Device architecture of the hybrid electrode/PEDOT:PSS (35 nm)/MAPbI_3_ (∼280 nm)/PCBM (∼60 nm)/Al (100 nm) cells tested in this study. (**b**) Cross-section SEM images of complete perovskite devices showed both Ag-mesh area (scale bar, 1 μm) and Flat PET area (scale bar, 500 nm). SEM top-view images of perovskite films; (**d**) : low-resolution image of perovskite film coated on both flat PET and Ag-mesh (scale bar, 2 μm); (**c**): film surface on bare PET (scale bar, 500 nm); (**e**): film surface on Ag-mesh (scale bar, 500 nm). (**f**) The corresponding energy-level diagram of each layer.

To build up flexible pero-SCs, we adopted a conventional p-i-n architecture in which the hybrid electrode is used as an anode, that is, PET/Ag-mesh/PH1000/poly(3,4-ethylenedioxythiophene):poly(styrenesulfonate) (PEDOT:PSS) (35 nm)/CH_3_NH_3_PbI_3_ (MAPbI_3_) (∼280 nm)/phenyl-C_61_-butyric acid methyl ester (PCBM) (∼60 nm)/Al (100 nm) as shown in Fig. 2a. This device structure offers the benefit of efficient charge transfer from the perovskite thin film to the charge transport layers, ensuring sufficient charge extraction^30^,^31^,^32^. The ∼57-μm-thick hybrid flexible electrode (PET/Ag-mesh/PH1000) and all organic interlayer materials (PEDOT:PSS and PCBM) ensure the ultrathin and flexible properties of pero-SCs. Notably, PH1000 and Ag-mesh in PET/Ag-mesh/PH1000 are used as electrode and transporting channel, respectively. PH1000 (∼0.9 S cm^−1^) can efficiently collect charges along both the lateral and vertical direction in less than 100 μm (ref. 33). The charges were then further gathered by the highly conductive Ag-mesh (>10^4^ S cm^−1^). As the periodic Ag-mesh honeycomb has a diagonal of 180 μm, the charge–transport pathway of PH1000 along the lateral direction is within 90 μm to ensure efficient charge extraction to the Ag mesh, and this is consistent with the low sheet resistance of the hybrid electrode. The perovskite films were fabricated by a standard two-step method (see Methods for details)^34^.

Cross-section scanning electron microscopy (SEM) images of an optimized device are presented in Fig. 2b. The MAPbI_3_ layer, with its distinct feature and contrast, can be well distinguished from the hole- and electron-transporting layers. The well-defined and pinhole-free perovskite film was observed from both the Ag-mesh area and the flat PET area, effectively depressing the shunting pathways. It is noteworthy to mention that the perovskite poly-crystalline structure can be grown in the Ag-mesh groove. Even though the depth of the groove is ∼400 nm after filling PH1000, the film quality remains. As shown in Fig. 2c–e, the top-view SEM images of the perovskite film further identified homogeneous MAPbI_3_ crystals in both the Ag-mesh and the flat PET area, confirming a high-quality film.

Figure 2f shows the energy band diagram of the pero-SC. The conduction band (CB) (3.75 eV) and the valence band (VB) (5.30 eV) in MAPbI_3_ are in good alignment with the LUMO level of PCBM (3.9 eV) and the work function of PEDOT:PSS (5.15 eV), respectively. Recent reports have alluded that minimizing the mismatch between the work functions of electrode and transporting layer can enhance charge transport through the transporting layer to its corresponding electrode^35^. Accordingly, we utilized scanning Kelvin probe microscopy to determine the surface potential (SP) difference of the PH1000 electrode before and after coating PEDOT:PSS. As shown in Supplementary Fig. 1b and f, in the flat surface, both the scanning Kelvin probe microscopy images of the PH1000 electrode before and after coating PEDOT:PSS are rather homogeneous. A slightly increased SP of 80 mV was obtained after coating PEDOT:PSS film, giving corresponding work function of 5.09 and 5.17 eV, respectively, which are rectified by the highly ordered pyrolytic graphite as standard sample^36^. Considering the similar work function and chemical compositions in PEDOT:PSS and PH1000, the contact should be Ohmic contact. These results indicate that there is no barrier for hole conduction from PEDOT:PSS to PH1000, which can efficiently avoid hole accumulation and facilitate greater transportation from PEDOT:PSS to PH1000. Moreover, in the Ag-mesh-covered area, the SP is not disturbed, and this further confirms the formation of continuous and homogeneous PEDOT:PSS films throughout the substrate surface.

### Performance of flexible pero-SCs

**Figure 3:**
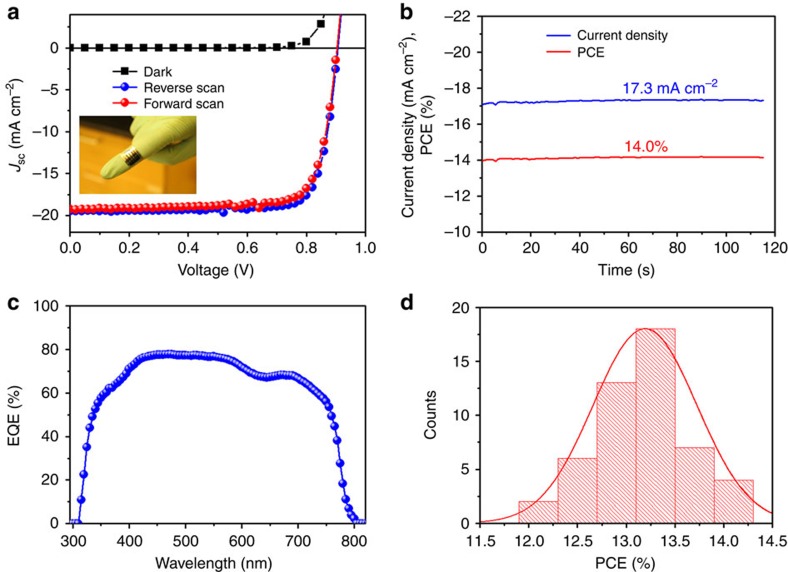
Photovoltaic performance characteristics. (**a**) *J*–*V* curves in reverse and forward scan measured under 100 mW cm^−2^ AM 1.5G illumination and dark for the champion flexible PET/Ag-mesh/PH1000/PEDOT:PSS/MAPbI_3_/PCBM/Al solar cell: Inset shows photograph of corresponding ultra-thin flexible pero-SCs. (**b**) Steady-state photocurrent output at the maximum power point (0.8 V). (**c**) External quantum efficiency (EQE) spectrum of champion flexible pero-SC. (**d**) Histograms of device PCE measured for 50 devices of PET/Ag-mesh/PH1000/PEDOT:PSS/MAPbI_3_/PCBM/Al.

Figure 3a shows the current density–voltage (*J*–*V)* characteristics of an inverted, planar, flexible PET/Ag-mesh/PH1000/PEDOT:PSS/perovskite/PCBM/Al solar cell. The champion flexible device achieved a power conversion efficiency of 13.7% with a short-circuit current (*J*_SC_) of 19.3 mA cm^−2^, open-circuit voltage (*V*_OC_) of 0.90 V, fill factor (FF) of 0.79 in the forward scan direction, and a PCE of 14.2% with a *J*_SC_ of 19.5 mA cm^−2^, *V*_OC_ of 0.91 V and FF of 0.80 in the reverse scan direction under 100 mW cm^−2^ AM 1.5G illumination. The flexible cell exhibits competitive efficiency as the control devices on rigid glass/ITO substrate as shown in Supplementary Fig. 2, indicating the enormous potential of our hybrid electrode in pero-SC. The slightly lower *J*_SC_ should be attributed to the lower transmission of the PET/Ag-mesh/PH1000 electrode (82–86% in the visible region) compared with that of glass/ITO electrode (82–91% in the visible region, Supplementary Fig. 3). The key photovoltaic parameters of the flexible and rigid planar perovskite solar cells are summarized in Supplementary Table 1. According to previous reports^17^,^18^, such p-i-n type devices usually show weak hysteresis with respect to the scan direction and rate owing to their fast response to an incident light signal^37^. In the case of flexible cells, the devices show even less hysteresis than the rigid ones. To accurately evaluate the efficiency, we took a steady-state current output at a forward bias of 0.8 V, which corresponds to the point of maximum power output, as shown in Fig. 3b. The flexible cell yielded a stabilized current density of 17.4 mA cm^−2^, corresponding to a power output of 14.0 mW cm^−2^, equal to 14.0% PCE. To the best of our knowledge, this is the highest PCE observed in a TCO-free flexible pero-SC. It is also noteworthy to mention that the flexible pero-SC gives a high specific power (the ratio of power to device weight) reaching 1.96 kW kg^−1^ owing to the ultrathin device, which also holds promising application in light-weight photovoltaic purpose, such as unmanned aerial vehicles^38^.

To accurately evaluate the hysteresis behaviours, the *J*–*V* curves of the optimizing devices based on PET/Ag-mesh/PH1000 and glass/ITO electrodes were measured at varied scan rates of 2.4 V s^−1^, 200 mV s^−1^ and 20 mV s^−1^ from both scanning directions as summarized in Supplementary Fig. 4 and Supplementary Table 2. In the case of flexible pero-SC, we confirm that the flexible pero-SC has less hysteresis than the control device, with slight change in *J*_SC_ in the range of 19.2–19.6 mA cm^−2^ and FF between 0.77 and 0.80. In contrast, the rigid pero-SCs showed obvious hysteresis. FF varied from 0.65 to 0.73 when the scanning rate is changed from 2.4 V s^−1^ to 200 mV s^−1^ in both scanning directions. Meanwhile, *J*_SC_ changes significantly from 21.1 mA cm^−2^ at high scan rate to 20.0 mA cm^−2^ at low scan rate. It indicates that it takes longer for the glass/ITO-based device to reach steady state. To confirm that, we measured photocurrent response at the maximum power point using manually controlled shutter (response time within 0.1 s) to turn on and off the incident light. As shown in Supplementary Fig. 4c and d, the photocurrent of flexible device rose quickly to maximum steady-state photocurrent. However, photocurrent from the rigid cell needs more than 1.9 s to fully saturate. Considering the similar structure of flexible and rigid devices, it is reasonable to attribute the less hysteresis of the flexible device to the high charge carrier extraction ability of hybrid electrode.

The external quantum efficiency spectra of the flexible pero-SC is shown in Fig. 3c. The integrated *J*_SC_ is 17.9 mA cm^−2^, close to the *J*_SC_ (19.5 mA cm^−2^) obtained under AM 1.5G solar simulator. The external quantum efficiency exceeds 70% from 400 to 610 nm, with a peak value of 78% at 520 nm. However, it drops to ∼68% in the long wavelength region of 630–700 nm, which should be owing to the incomplete absorption of light. The perovskite layer is relatively thin, and the absorption coefficient is also low in this range. As the reproducibility of device efficiency has been a concern of the scientific community, we further show a histogram of the PCE of the flexible pero-SCs in Fig. 3d. Over 50 devices were fabricated and characterized, producing a 4.0% relative standard deviation in PCE, and thus the PCEs and reproducibility are also encouraging.

**Figure 4:**
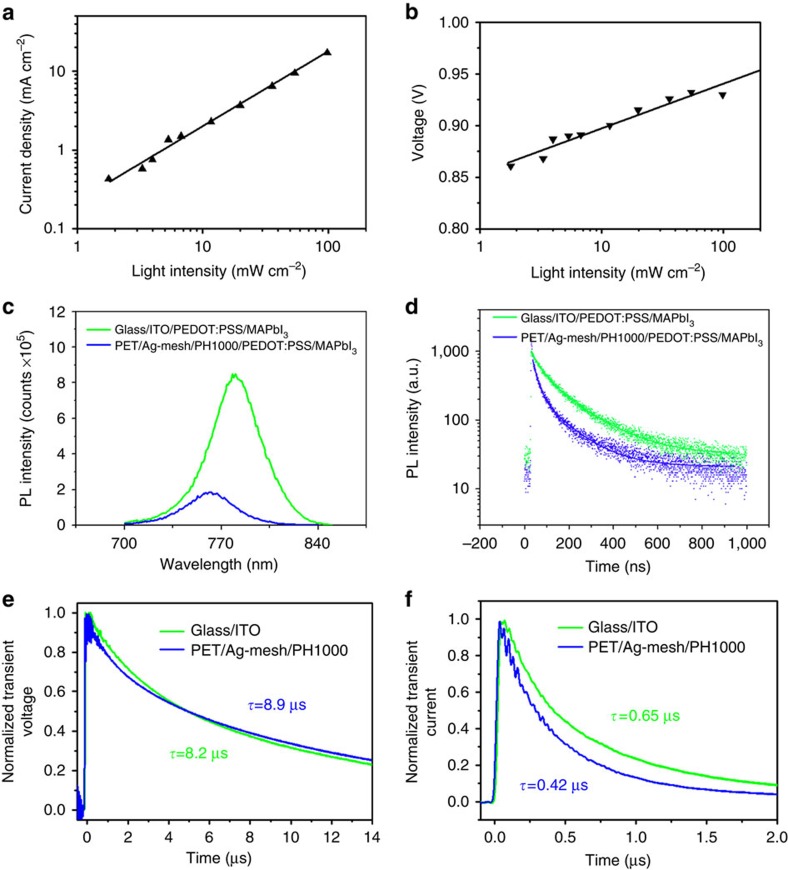
Characterization of flexible pero-SCs. (**a**) *J*_SC_ as a function of light intensity in a double log plot. (**b**) *V*_OC_ as a function of light intensity in a semi-log scale. (**c**) The steady-state PL spectra of perovskite film on various substrates, and (**d**) TRPL decay transient spectra of PET/Ag-mesh/PH1000/PEDOT:PSS/MAPbI_3_ and glass/ITO/PEDOT:PSS/MAPbI_3_. Charge transient time and traces for optimized devices based on glass/ITO and PET/Ag-mesh/PH1000 electrodes by (**e**) transient photovoltage measurement under 0.5 sun bias light and (**f**) transient photocurrent measurement.

To further understand the device operation in the flexible pero-SCs, light intensity dependence of *J*–*V* characteristics of the device was measured under illumination of a solar simulator with a set of neutral density filters. Figure 4a shows the linear relationship of the photocurrent with light intensity in a double logarithmic scale for the device with a slope close to unity, indicating no substantial space charge build-up and confirming the good electrical contact in the device^39^. Figure 4b gives *V*_OC_ as a function of light intensity. We can see that *V*_OC_ increases monotonically with light intensity for the flexible pero-SCs. The dependence of *V*_OC_ on light intensity implies that trap-assisted Schockley–Read–Hall recombination plays a minor role even at low light intensity^40^.

We then looked into the charge dynamics by measuring steady-state photoluminescence (PL) and time-resolved photoluminescence (TRPL) characterization. Both have been recognized to be proper tools to understand the charge extraction in perovskite-based solar cells. Figure 4c shows the steady-state PL spectra of MAPbI_3_ films on PET/Ag-mesh/PH1000/PEDOT:PSS and glass/ITO/PEDOT:PSS. The PL intensity on PET/Ag-mesh/PH1000/PEDOT:PSS was reduced by one order of magnitude compared with that on glass/ITO/PEDOT:PSS. It strongly indicates that this PET/Ag-mesh/PH1000 hybrid electrode coated with PEDOT:PSS hole-transporting layer can extract charge carrier more efficiently, which is consistent with the less hysteresis of the flexible pero-SCs. The TRPL was measured by monitoring the peak emission at 768 nm as shown in Fig. 4d. Fitting the data with bi-exponential decay yields a fast decay (*τ*_1_) component and a slow decay (*τ*_2_) component, and the detailed parameters are summarized in Supplementary Table 3. The fast decay would be originated from the quenching of charge carriers by the anode contact, and the slow decay component could be attributed to the radiative recombination of free charge carriers before the charge collection^41^. In the case of glass/ITO/PEDOT:PSS/MAPbI_3_, the fast decay lifetime was 43.67 ns with a 61.3% ratio and the slow decay lifetime was 165.95 ns with a 38.7% ratio, suggesting that the depopulation of photogenerated charges was dominated by charge collection through the PEDOT:PSS/perovskite interface and appropriate radiative recombination. For PET/Ag-mesh/PH1000/PEDOT:PSS/MAPbI_3_, both *τ*_1_ and *τ*_2_ were shortened to 22.34 and 129.40 ns, respectively, and *τ*_1_ dominated the PL decay, which can be interpreted that the hybrid PET/Ag-mesh/PH1000 electrode induces a faster charge transfer from MAPbI_3_. Therefore, it is apparent that the majority of photogenerated carriers were collected by the electrode with minimal recombination loss. The fast charge transfer and efficient collection shown in the devices based on PET/Ag-mesh/PH1000/PEDOT:PSS/MAPbI_3_ may be closely related to the less interfacial barrier and a more efficient charge carrier extraction as discussed above, thus contributing to an enhancement of photovoltaic properties, such as less hysteresis and high FF.

We then further study the effects of the flexible hybrid electrode on the charge carrier recombination and transport characteristics in complete devices by measuring the charge carrier lifetime and charge carrier extraction time via transient photovoltage (TPV) and transient photocurrent (TPC) methods, respectively. As shown in Fig. 4e, the carrier lifetime of flexible device was slightly increased to 8.9 μs, compared with 8.2 μs in glass/ITO-based device, under 0.5 sun bias light at open-circuit condition, suggesting the similar perovskite film quality on both flexible and rigid substrates. In Fig. 4f, the photocurrent decay time at short circuit condition decreased to 0.42 μs for the flexible device, compared with 0.65 μs for the rigid device. The high charge carrier extraction capability of PET/Ag-mesh/PH1000 electrode reduced charge carrier extraction time by 1.5 times, thus benefiting for the efficient charge carrier separation and extraction.

**Figure 5:**
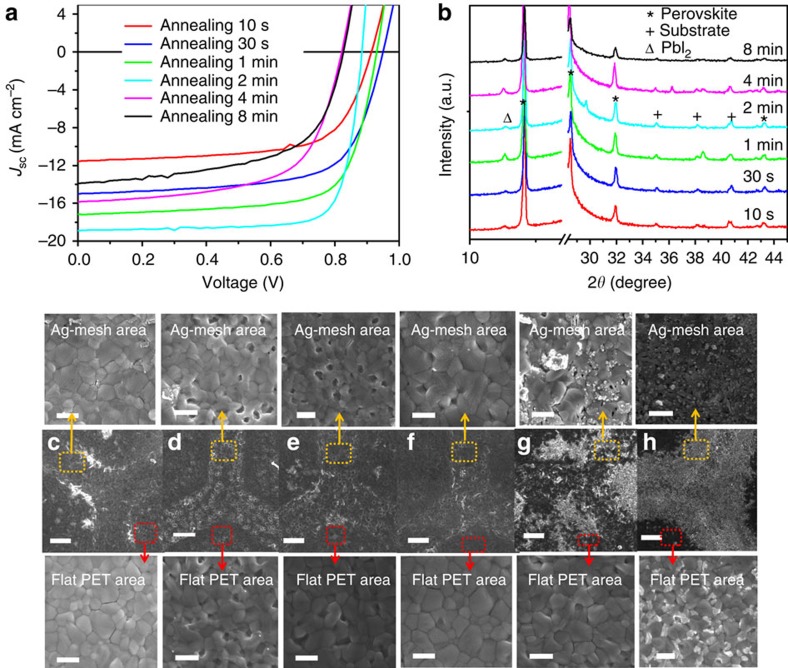
Annealing-time evolution of the perovskite films. Annealing MAPbI_3_ grown on PET/Ag-mesh/PH1000/PEDOT:PSS substrate at 130 °C for 10 s, 30 s, 1 min, 2 min, 4 min and 8 min, respectively: (**a**) *J*–*V* curves of the corresponding solar cells. (**b**) X-ray diffraction patterns of the perovskite films. (**c**–**h**) Top-view SEM images of the perovskite films coated on both flat PET and Ag-mesh area (scale bar, 2 μm): (**c**) 10 s; (**d**) 30 s; (**e**) 1 min; (**f**) 2 min; (**g**) 4 min; (**h**) 8 min. Ag-mesh area row: the SEM images of corresponding perovskite films coated on Ag-mesh (scale bar, 500 nm); Flat PET area row: SEM images of corresponding perovskite films coated on flat PET (scale bar, 500 nm).

One concern regarding the Ag-mesh electrode is that Ag may react with the halogen in the perovskite film, which is likely owing to the formation of a silver halide, especially when annealed in ambient air. When we deposited MAPbI_3−*x*_Cl_*x*_ film via the one-step method, which requires thermal annealing for ∼1.5 h, it was found that the long thermal annealing process can dramatically decrease the conductivity of the embedded Ag-mesh and the corresponding device performance (Supplementary Fig. 5). We attribute this to the formation of non-conducting silver halide. Therefore, we used the two-step deposition method to avoid long-time thermal annealing, which results in the reaction between Ag-mesh and perovskite and also balanced the growth of perovskite crystal on the substrate consisting of double components (flat PET and Ag-mesh groove). On the PET/Ag-mesh/PH1000 substrates, we found that there was a strong dependence of photovoltaic performance on thermal annealing as shown in Fig. 5a and Supplementary Table 1. When the annealing time was extended from 10 s to 2 min at 130 °C, the PCEs improved from 7.0% to 13.1%. With further thermal annealing up to 8 min, the PCEs dramatically dropped to 6.8%. We then studied the relationship between photovoltaic performance and thermal annealing time, and the composition/morphology evolution of two-step solution-processed MAPbI_3_ crystals on PET/Ag-mesh/PH1000 substrate was further investigated by using SEM and X-ray diffraction. Films with different annealing times (10 s, 30 s, 1 min, 2 min, 4 min and 8 min) were chosen for the X-ray diffraction patterns and SEM images shown in Fig. 5. As shown in Fig. 5b, the perovskite film on the PET/Ag-mesh/PH1000 substrate, after quick annealing for 10 s, exhibited clear diffraction peaks at 14.1°, 28.5° and 31.9°, and were assigned to the (100), (200) and (210) planes, respectively, characteristic of a cubic perovskite structure^42^. With increasing annealing time, the diffraction peaks remain, which indicates that the major part of the precursors was converted to MAPbI_3_ crystal even after short-time annealing at 130 °C. Nevertheless, residual PbI_2_ is present according to the X-ray diffraction pattern, which is favourable for pero-SC via surface passivation^43^. Moreover, as illustrated in Supplementary Fig. 6a, the full width at half maximum of the (100) peak reduce gradually from 0.203° to 0.178° when the thermal annealing time increased from 30 s to 8 min. It should be noted that both increased crystallinity and a larger grain size with less grain boundaries are evidenced by narrowed X-ray diffraction peak, which is strongly related to the photovoltaic performance^44^. However, films annealed for 4 and 8 min exhibit higher crystallinity, but dramatically decreased PCEs, compared with optimized condition. It is probably caused by decomposition of perovskite film and reaction between perovskite and Ag-mesh as discussed below.

SEM images (Fig. 5c–h) further reveal the morphology evolution of the perovskite films upon annealing. The perovskite film (Fig. 5c) was compact and conformal on top of both the PET and the Ag-mesh after thermal annealing for 10 s. On the edge of the Ag-mesh, we observed relatively high brightness contrast compared with the adjacent area, possibly owing to less conductivity and more accumulation of charges. Precursor conversion is not complete, likely owing to the non-uniform thermal conductivity of the hybrid electrode, considering the higher thermal conductivity of Ag-mesh than those of the polymer and conducting polymer components. With the annealing time increased to 30 s, the appearance of pinholes was observed. Again the change in film morphology, along with improved photovoltaic efficiency, (that is, dramatic *J*_SC_ enhancement and reduction in series resistance) indicates that the conversion of precursors into perovskite is still favoured. As for the pinholes, we coated a PCBM layer to fully cover the perovskite film surface and thus prevent shunting owing to direct contact between Al cathode and PEDOT:PSS layer^41^,^45^. Increasing the annealing time to 1 and 2 min, the film morphology was further improved, giving a continuous and homogeneous surface with larger grain size and a compact perovskite film. The optimal morphology of the perovskite film can facilitate the charge carrier transportation and collection, and can further contribute to an improved PCE value. Further increasing the annealing time to 4 and 8 min, however, results in a large number of bright contrast disordered grains, especially around the Ag-mesh, strongly indicating decomposition of the perovskite films owing to overheating. The photovoltaic cells based on these films yielded inferior PCEs below 10%. Compared with the optimized devices, all the three parameters determining PCE, that is, *V*_OC_, *J*_SC_ and FF, decreased. It was further found that long-time annealing at 130 °C will result in the decomposition of the perovskite films.

To understand the correlation between charge carrier extraction dynamics and crystallographic properties of perovskite films, we performed TPV and TPC measurement as summarized in Supplementary Fig. 6 and Supplementary Table 1. According to the TPV data, extending the annealing time from 1 to 2 min increased carrier lifetime from 5.3 to 8.2 μs, which can be ascribed to the fewer defects in the perovskite film consistent with the increased crystallinity of perovskite films and larger grain size. With further annealing up to 4 and 8 min, the carrier lifetime dramatically decreased to 3.4 and 1.7 μs, respectively, which, as we discussed in previous section, may result from perovskite decomposition. From TPC measurement, the 2 min annealed device has the fastest charge extraction, which is consistent with continuous and homogeneous film surface, large grain size, high crystallinity and beneficial to the device performance, especially FF and *J*_SC_. However, after annealing up to 4 and 8 min, charge carrier extraction times are significantly increased. It could be caused by more defects in the perovskite film, resulting from the decomposition of the perovskite films and reaction between Ag-mesh and perovskite because of overheating. Therefore, the high device performance is likely owing to the high perovskite crystallinity with less defects and well protected Ag-mesh.

### Bending and thermal stability test of flexible pero-SCs

**Figure 6:**
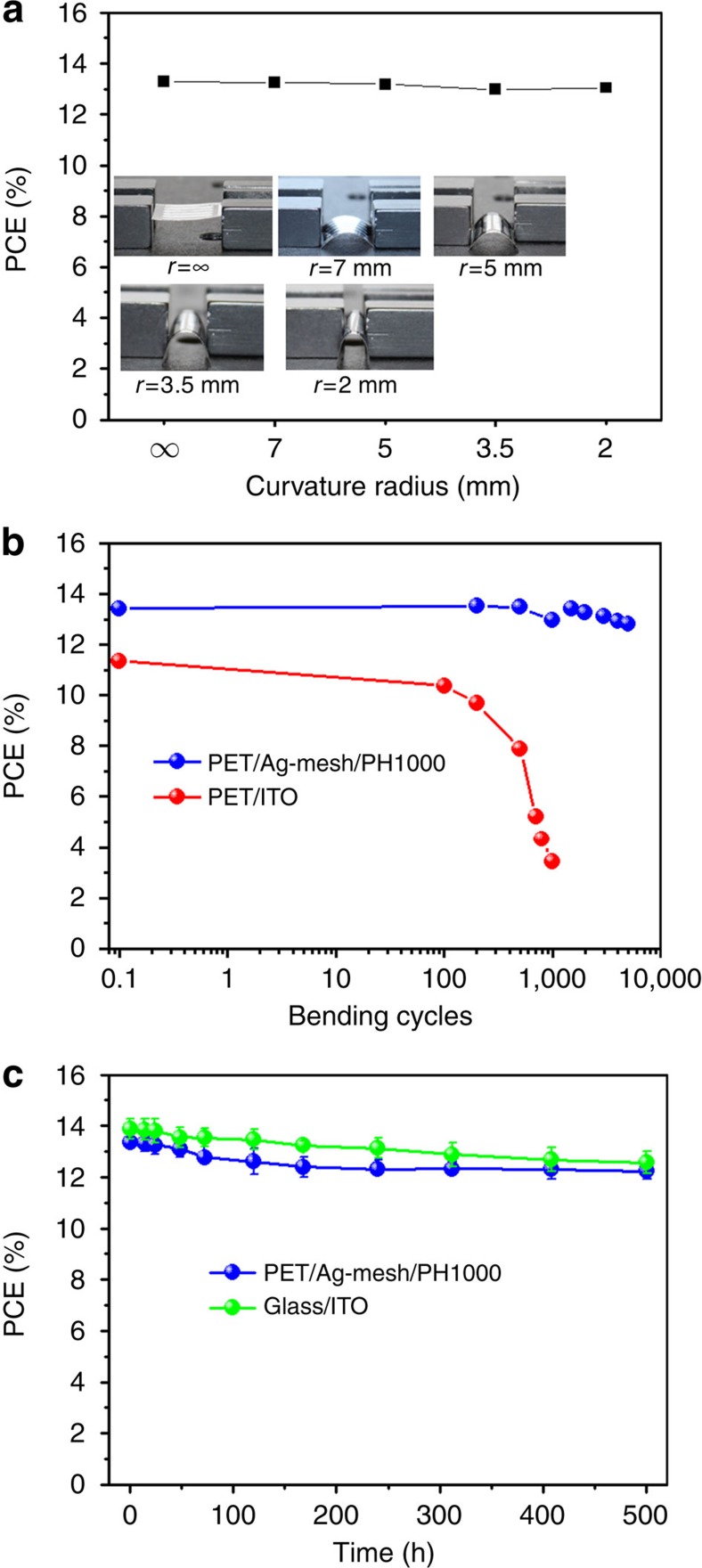
Bending and stability test of flexible pero-SCs. (**a**) PCEs measured after bending PET/Ag-mesh/PH1000 electrode-based flexible pero-SCs within a specified radius of ∞, 7, 5, 3.5 and 2 mm. The inset shows the real images of the corresponding bending radii, respectively. (**b**) PCEs of flexible pero-SCs based on both PET/Ag-mesh/PH1000 and PET/ITO electrodes as a function of bending cycles at a radius of 5 mm. (**c**) Stability of pero-SCs based on both PET/Ag-mesh/PH1000 and glass/ITO substrates under room temperature in N_2_-filled glove box in a timescale of a few hundred hours. PCE values are obtained from statistical distribution of six devices for each condition. The ball symbols represent the mean, while the line across the ball represents the distribution.

From the viewpoint of the utilization of the flexible pero-SCs, the mechanical flexibility and durability under bending stress are of great importance concerning practical applications, such as portable and wearable electronics. Bending tests, taking into account the bending radii and cycles, were carried out to evaluate the device stability against mechanical bending. The flexible pero-SCs were bent with five different radii of curvature (*r*=∞, 7, 5, 3.5 and 2 mm) in one bending cycle as shown in Fig. 6a inset. The PCE showed almost no decrease in efficiency even with 2 mm of bending radius, which is closing to the limit bending radius, retaining 98.1% of the original PCE value (Fig. 6a). This result indicates that both the PET/Ag-mesh/PH1000 electrode and perovskite film possess high flexibility. We further evaluated the effects of mechanical bending on PET/Ag-mesh/PH1000 electrode via multiple-cycles bending test. A reference pero-SC on PET/ITO electrode was also fabricated for comparison. A total of 5,000 consecutive bending cycles at radius of 5 mm were conducted. As shown in Fig. 6b, the flexible pero-SCs based on PET/Ag-mesh/PH1000 electrode display very promising mechanical bending stability. The PCE sustained even after 1,500-cycle bending. We then further went up to 5,000 cycles, the device still retaining 95.4% of its initial PCE, accompanying the well-maintained key photovoltaic parameters such as *J*_SC_, *V*_OC_ and FF (Supplementary Fig. 7). Such high bending durability is very competitive to the state-of-the-art in flexible solar cell field, while still holding the advantage of high efficiency^46^. The reference on PET/ITO substrate showed obviously deteriorated trend of device performance after 100 bending cycles, consistent with previous report^20^. PCE decreased to 30% of its initial value after 1,000 bending cycles. As reported by Jung and colleagues^20^, the sheet resistance of PET/ITO electrode abruptly increased owing to the fracture in ITO polycrystalline structure, which results in the dramatic deterioration in device performance. Therefore, our results demonstrated that the high flexibility and durability of pero-SCs on flexible hybrid substrates are ascribed to the combination of embedded Ag-mesh charge transport channel in a PET substrate and high conductivity polymer electrode. The former reserved the characteristic of high mechanical flexibility and durability of PET substrate owing to the low coverage and embedded Ag-mesh. The latter utilized the high flexibility nature of the polymer. More importantly, we proved that the inorganic–organic halide perovskites possessed the high flexibility and promising durability, which can further extend its state-of-the-art flexible electronics expectation.

The mechanical deformation caused ∼5% loss in PCE after 5,000 bending cycles, stimulating us toward more insight into bending durability of pervoskite film. As illustrated in Supplementary Fig. 8e, the perovskite film retains the cubic structure with the same full width at half maximum after bending 2,000 and 5,000 cycles, indicating that the crystallinity does not degrade upon bending. Meanwhile, 2,000 bending cycles does not cause any observable changes in film morphology (Supplementary Fig. 8b). Similarly, the perovskite film after 5,000 bending cycles shows no cracks from both top and cross-section view as shown in Supplementary Fig. 8c and d. However, after 5,000 bending cycles, as shown in higher-resolution SEM image (Supplementary Fig. 8c), some pinholes with diameter of 20–50 nm were observed close to the grain boundaries. The developed pinholes could be responsible for the efficiency loss. Steady-state PL and TRPL measurement of the bent-state perovskite film were used to further investigate the issue, and the data are shown in Supplementary Fig. 9 and Supplementary Table 4. The PL intensities of perovskite film before and after bending are almost identical, indicating that the electronic coupling between layers in device is not affected by the bending process, and the flexible electrode coated with PEDOT:PSS hole-transporting layer can still extract charge carrier efficiently even after 5,000 bending cycles. The fast component of PL decay, *τ*_1_, were also similar according to TRPL data. However, with increasing bending cycles to 5,000, the slow component, *τ*_2_, slightly decreases, and the ratio of *τ*_2_ in the overall decay process increases. As *τ*_2_ comes from the delayed recombination of trapped charges in perovskite bulk film, the pinholes originating from the mechanical bending could be the reason of slightly increased trap density and thus the decrease in photovoltaic efficiency.

Device thermal stability is always another major concern with regard to the potential of hybrid electrodes. In particular, solar cells normally operate at elevated temperatures. Therefore we recorded the device efficiency as a function of time at various temperatures. The flexible pero-SC stored in inert atmosphere is relatively stable at room temperature as illustrated in Fig. 6c. PCE of 12.2% retains 91.6% of initial value after 500 h storage, which is slightly better than that of the rigid control device (90.5% of initial PCE). At 45 and 70 °C, a similar trend holds as shown in Supplementary Fig. 10. The efficiency of flexible device decreased by ∼25% (PCE of 9.60%) after 500 h at 45 °C, while the rigid device lost ∼31% (PCE of 9.45%). At 70 °C, both flexible and rigid pero-SCs deteriorated quickly and showed photovoltaic characteristics with ∼23 and 15% of their initial values, respectively, after 97 h. It could be attributed to the intrinsic issues of this device structure, such as the acidic PEDOT:PSS, crystallization of PCBM, as well as the thermal stability of perovskite film^47^. According to our results, the flexible electrode does not induce noticeable device degradation at the initial stage of 500 h of stability test. Therefore, the PET/Ag-mesh/PH1000 hybrid electrode is a promising candidate for large-scale manufacturing of pero-SCs.

## Discussion

In summary, ultrathin flexible pero-SCs with planar heterojunction architecture have been successfully constructed on 50-μm-thick PET substrate-based Ag-mesh/PH1000 hybrid electrode. A currently highest PCE (14.0%) of TCO-free flexible pero-SCs has been reported. The keys to achieve highly efficient flexible pero-SCs are the high transmission, low sheet resistance, excellent thermal stability of this hybrid electrode, as well as the well-designed device architecture with aligned work functions of PH1000/PEDOT:PSS efficiently extracting charge carriers, and a perfect perovskite film. The ultrathin flexible pero-SCs exhibit excellent bending durability retaining over 95.4% of its initial PCE value even after 5,000 fully bending cycles. This study opens an effective protocol for fabricating efficient, high specific power and bending durable flexible pero-SCs.

## Methods

### FEAM substrate fabrication

The fabrication process of FEAM is described as follows. First, the bottom of PET (thickness of 50 μm) substrate was covered by a highly hardening treatment PET protection film (thickness of 100 μm), which can well prevent distortion of the PET substrate during device fabrication process and be easily peeled from the FEAMs-based device. Second, we design and fabricate a protruding hexagon-patterned nickel (Ni) mould (diagonal length of 90 μm, width of 3 μm and height of 2 μm) through electroforming. Then the Ni mould with an area 500 mm × 630 mm was wrapped around a steel roller with the diameter of 200 mm, forming Ni roller mould. The ultraviolet-resin (thickness of 7 μm)-coated PET (thickness of 50 μm) substrate was transferred at a speed of 10 m min^−1^ by roll-to-roll nanoimprinting machine, and imprinted by the Ni roller mould with a pressure of 0.25 g under ultraviolet irradiation. The roll-to-roll ultraviolet nanoimprinting lithography can significantly improve the substrate imprinting efficiency. Third, the imprinted groove was filled with Ag ink containing conductive submicron Ag nanoparticles (viscosity of 500 c.p.s.) and the excess Ag ink was removed by means of scraping. The same process was repeated at least three times for complete filling. Meanwhile, to make the surface clear, the wiping process was performed with ethanol. Subsequently, the substrate underwent sintering at 80 °C for 10 min. Finally, an antistatic protective film was covered on the completed substrate.

### Flexible pero-SCs fabrication

A 15-inch FEAM substrate containing 100 pieces of modules with a size of 15 mm × 15 mm were cut off and then peeled off the antistatic protective film, on which we fabricated perovskite solar cells, directly. The processing and device geometry are exactly the same as the reference cells made on the ITO-coated glass. The Supplementary Fig. 11 showed the fabricated process of flexible pero-SC as the following steps: Step 1, the high conductivity polymer PH1000 (Heraeus, Clevios PH1000, Germany) with the thickness of 150 nm was spin-coated on the surface of FEAM under 800 r.p.m., and then baked at 120 °C for 15 min in ambient air. Step 2, an ∼40-nm-thick buffer layer of PEDOT-4083 (Heraeus, Clevios PVPAI 4083, Germany) was subsequently spin-coated at 2,000 r.p.m. and baked at 120 °C for 20 min in ambient air. Step 3, the substrates were transferred into nitrogen glove box for coating of the perovskite layer. In this study, for perovskite layer, we adopted two-step spin-coating method to make a dense and highly uniform film. The 1 M PbI_2_ solution was stirred in *N*,*N*-Dimethyl formamide solvent at 70 °C for 4 h. The PbI_2_ solution was spin-coated on substrates and then dried at 70 °C for 10 min. Step 4 and 5, 50 mg ml^−1^ of CH_3_NH_3_I (MAI) in 2-propanol solution was coated on PbI_2_ layer and the film was taken out for annealing in ambient air at 130 °C for 1 min 40 s for the champion device. Step 6, for PCBM coating (∼60 nm), a 2 wt% PCBM in chlorobenzene solution was spin-coated onto the perovskite layer. Step 7, the devices were transferred into a vacuum chamber for 100 nm Al electrode deposition by thermal evaporation at 4 × 10^−6^ bar with a shadow mask. The active area was 0.1 cm^2^. Finally, step 8, the highly hardening treatment PET protection film coated on the bottom of FEAM substrate was peeled off. The ultra-thin flexible pero-SCs were obtained. The detailed device and perovskite film for characterization are described in Supplementary Methods.

## Additional information

**How to cite this article:** Li, Y. *et al*. High-efficiency robust perovskite solar cells on ultrathin flexible substrates. *Nat. Commun.* 7:10214 doi: 10.1038/ncomms10214 (2016).

## Supplementary Material

Supplementary Information Supplementary Figures 1-11, Supplementary Tables 1-4 and Supplementary Methods
