# Supplementary material for: High-efficiency robust perovskite solar cells on ultrathin flexible substrates
Source: Nat Commun. 2016 Jan 11;7:10214. doi: 10.1038/ncomms10214 (PMC4729901; doi:10.1038/ncomms10214)
Supplement: Supplementary Information — Supplementary Figures 1-11, Supplementary Tables 1-4 and Supplementary Methods [file ncomms10214-s1.pdf]

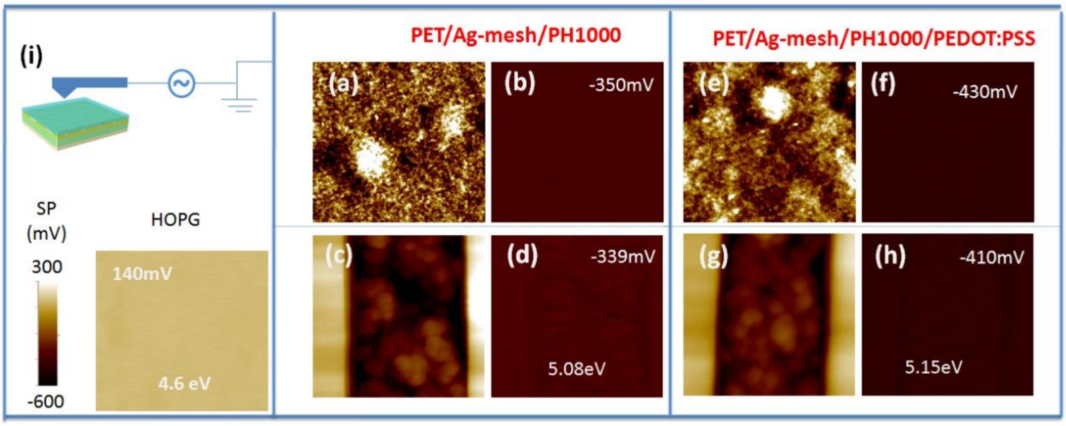

Supplementary Figure 1. The AFM height and SKPM images of PET/Ag-mesh/PH1000 and PET/Ag-mesh/PH1000/PEDOT:PSS substrates. (a, e) AFM height images on the flat PET area. (c, g) AFM height images on Ag-mesh area. (b, f) SKPM images on the flat PET area. (d, h) SKPM images on the Ag-mesh area.

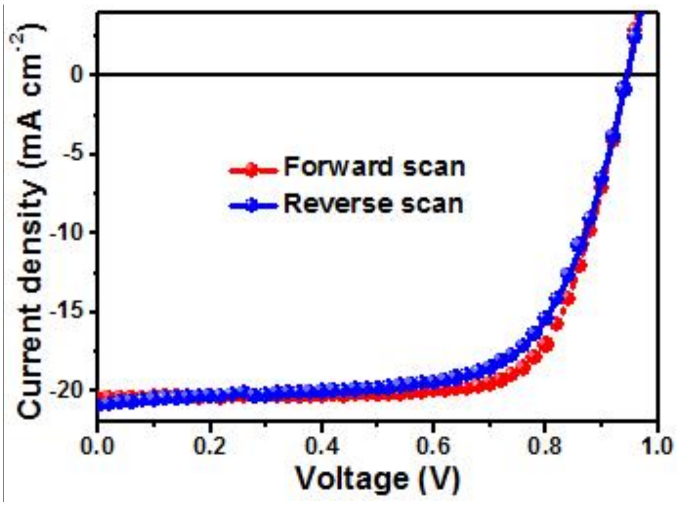

Supplementary Figure 2. *J*-*V* curves in reverse and forward scan measured under 100 mW cm^{-2} AM 1.5G illumination for the Glass/ITO/PEDOT:PSS/$MAPbI_3$/PCBM/Al solar cell.

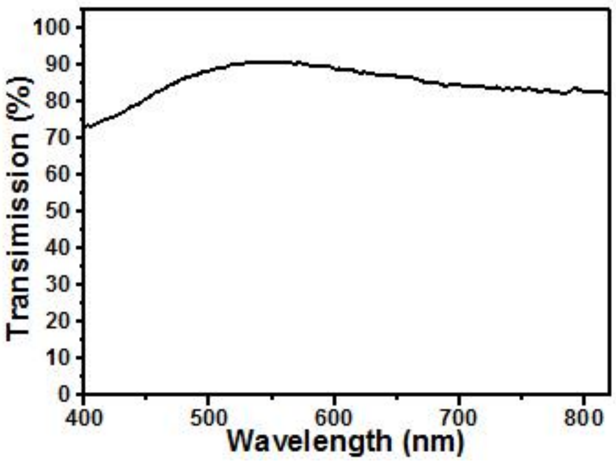

Supplementary Figure 3. Transmission spectrum of Glass/ITO substrate.

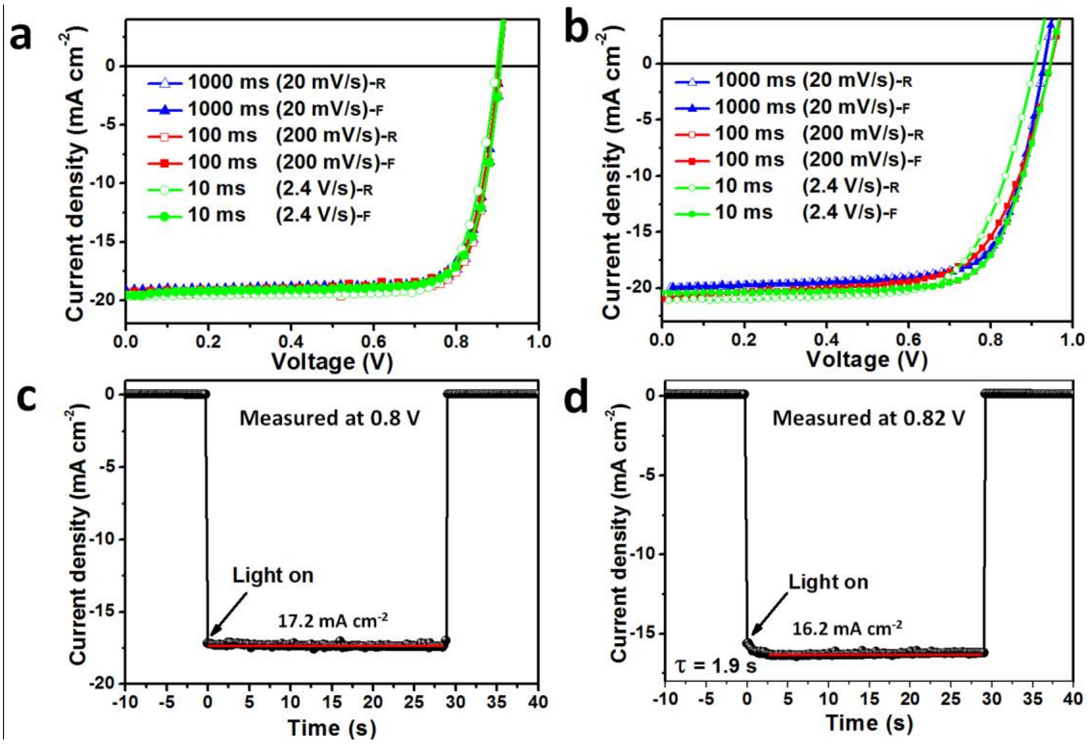

Supplementary Figure 4. *J*-*V* curves of optimized devices at different voltage scan-rates and scanning direction (R: reverse scan direction, F: forward scan direction) in voltage delay time (ms) (a) for PET/Ag-mesh/PH1000 electrode and (b) for Glass/ITO electrode based pero-SCs. Photocurrent response process on turning on and off the incident light at the maximum power point: (c) for PET/Ag-mesh/PH1000 electrode measured at 0.8 V and (d) for Glass/ITO electrode based pero-SCs measured at 0.82 V.

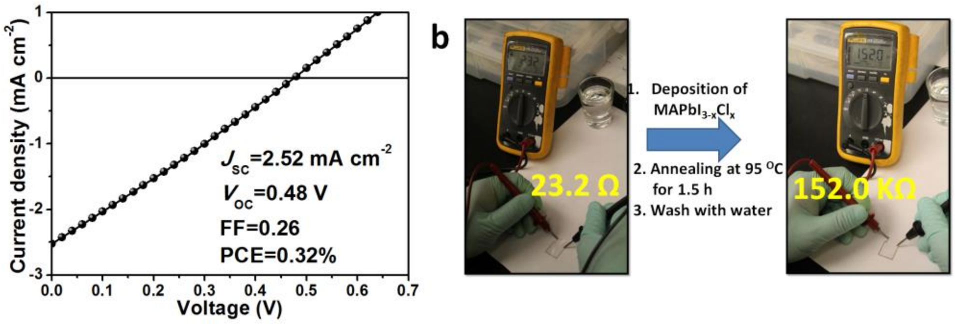

Supplementary Figure 5. (a) The *J-V* curve of flexible pero-SC based on PET/Ag-mesh/PH1000/$MAPbI_3$/PCBM/Al by annealing in ambient air at 95 ^oC for 1.5 h. (b) the photographs of resistance test before and after annealing process.

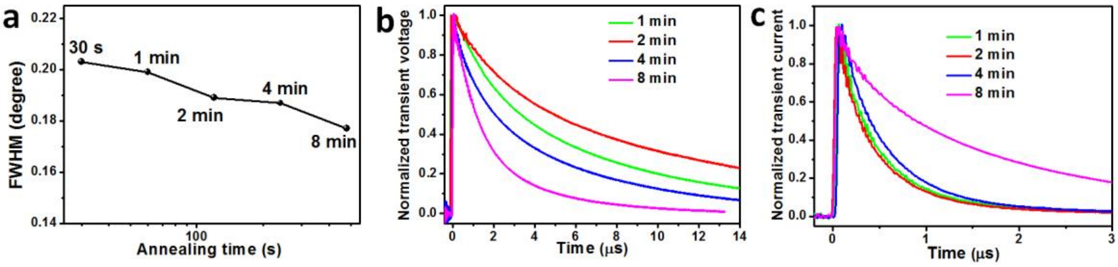

Supplementary Figure 6. (a) FWHM evolution of the (100) diffraction peak from XRD patterns of the perovskite films coated on PET/Ag-mesh/PH1000 electrode with an increased annealing time. Charge transient traces for the devices based on PET/Ag-mesh/PH1000 electrodes with different thermal annealing times by (b) transient photovoltage measurement under 0.5 sun bias light and (c) transient photocurrent measurement.

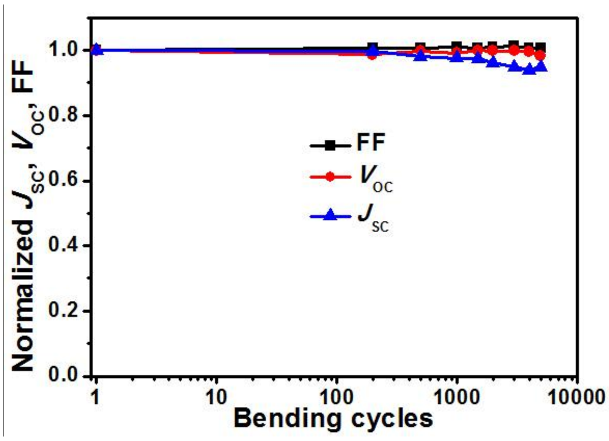

Supplementary Figure 7. Normalized photovoltaic parameters (J_{SC}, V_{OC}, FF) of flexible pero-SCs based on PET/Ag-mesh/PH1000 electrode as a function of bending cycles at radius of 5 mm.

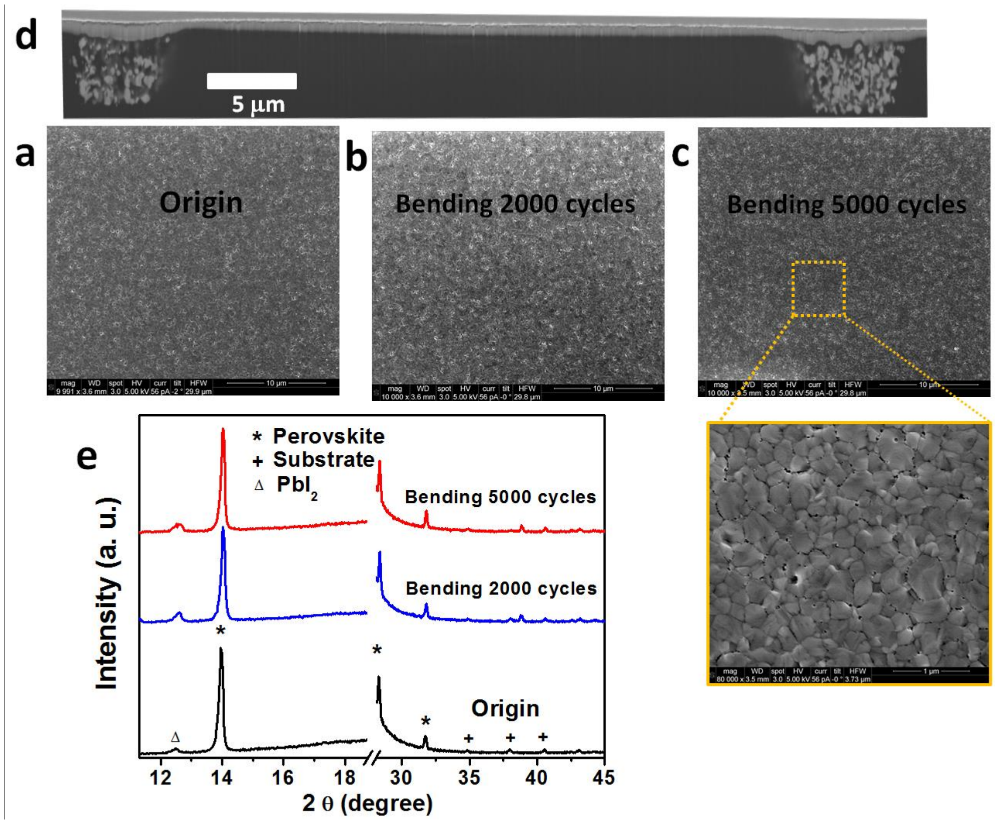

Supplementary Figure 8. SEM top-view images of perovskite films in low-magnification (a) origin; (b) after 2000 bending cycles at radius of 5 mm; (c) after 5000 bending cycles at radius of 5 mm and its corresponding image with high-magnification. (d) Cross-section SEM image of complete perovskite device with 5000 bending cycles at radius of 5 mm in low-magnification. (e) XRD patterns of perovskite film with different bending cycles at radius of 5 mm.

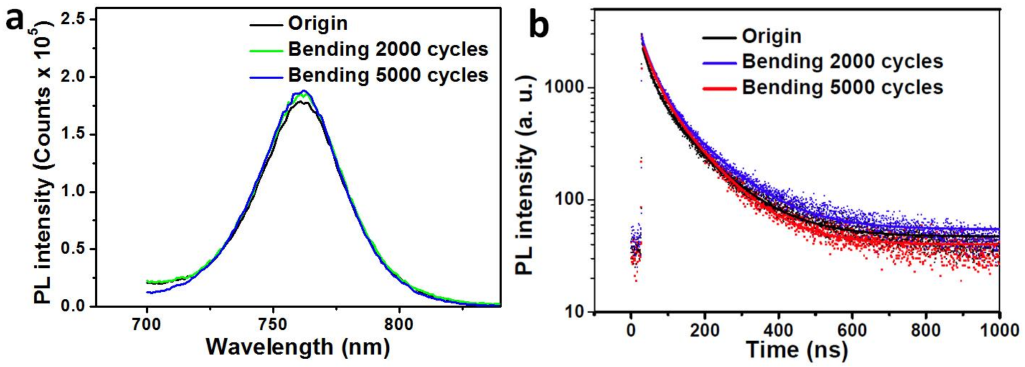

Supplementary Figure 9. (a) The steady-state PL spectra and (b) TRPL decay spectra of perovskite film on PET/Ag-mesh/PH1000/PEDOT:PSS with varied bending cycles at radius of 5 mm.

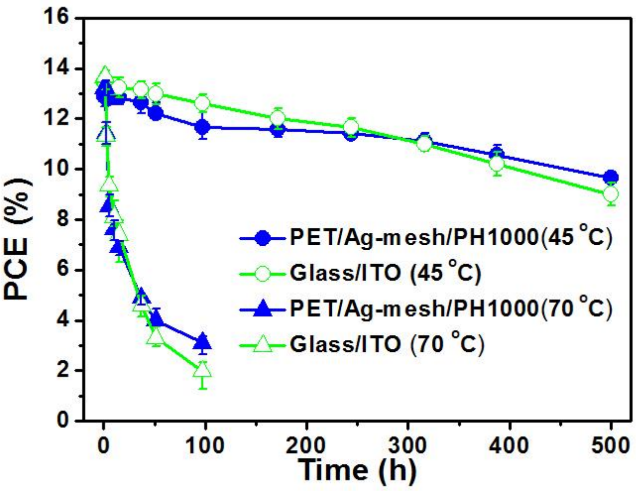

Supplementary Figure 10. Stability test of pero-SCs based on both PET/Ag-mesh/PH1000 and Glass/ITO substrates under different temperature in N_2-filled glove box in a time scale of a few hundred hours. PCE values are obtained from statistical distribution of 6 devices for each condition. The ball symbols represents the mean, while the line across the ball represents the distribution.

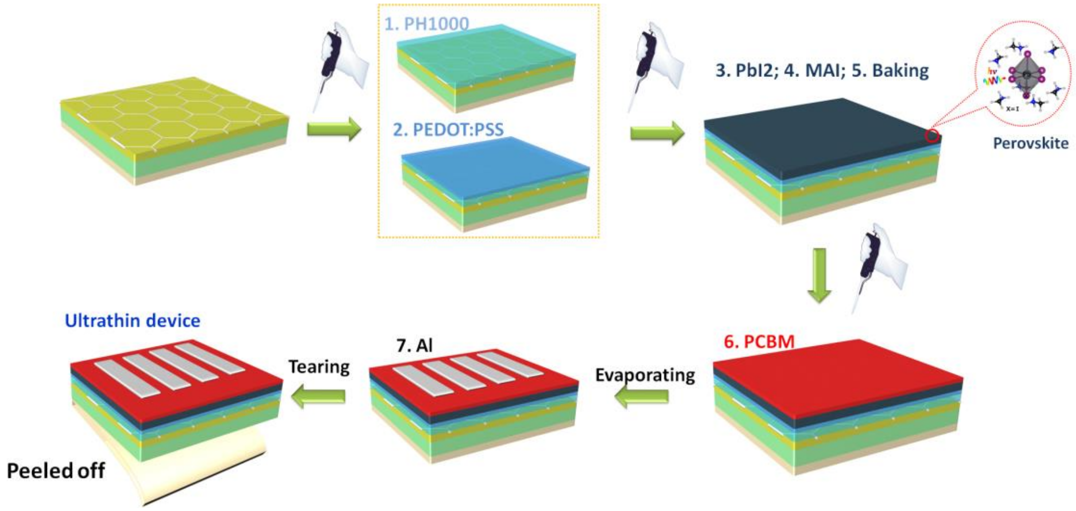

Supplementary Figure 11. The fabricated process of ultrathin flexible pero-SC.

Supplementary Table 1. Summary of device performance of the pero-SCs constructed on the PET/Ag-mesh/PH1000 and Glass/ITO electrodes.

Device	Annealing time	V_{OC} (V)	FF (%)	J_{SC} (mA cm^{-2})	PCE (%)	Transient current decay (μs)[a]	Transient voltage decay (μs)[a]
Flexible-champion (Forward scan)	1 min 40s	0.90	0.79	19.3	13.7	--	--
Flexible-champion (Reverse scan)	1 min 40s	0.91	0.80	19.5	14.2	--	--
Glass/ITO-PVSK (Forward scan)	1 min 40s	0.95	0.65	20.9	12.9	--	--
Glass/ITO-PVSK (Reverse scan)	1 min 40s	0.94	0.73	20.5	0.94	--	--
Flexible -10s	10 s	0.91	0.66	11.6	7.0	--	--
Flexible -30s	30 s	0.95	0.67	15.0	9.6	--	--
Flexible -1m	1 min	0.93	0.71	17.2	11.4	0.47	5.3
Flexible -2m	2 min	0.88	0.78	18.9	13.1	0.42	8.2
Flexible -4m	4 min	0.82	0.63	15.8	8.2	0.57	3.4
Flexible -8m	8 min	0.83	0.59	13.9	6.8	1.48	1.7

Forward scan: 0 V→1.2 V, scan-rate 200 mV s^{-1}, delay time 100 ms; Reverse scan: 1.2 V→0 V, scan-rate 200 mV s^{-1}, delay time 100 ms.

Supplementary Table 2. Summary of optimized device performance at different voltage scan-rates and scanning direction for the Glass/ITO based pero-SC with structure of Glass/ITO/PEDOT:PSS/$MAPbI_3$/PCBM/Al and flexible pero-SCs with structure of PET/Ag-mesh/PH1000/$MAPbI_3$/PCBM/Al.

Device	V_{OC} (V)	FF (%)	J_{SC} (mA cm^{-2})	PCE (%)
Glass/ITO-PVSK (20 mV s^{-1})-R	0.93	0.72	20.0	13.4
Glass/ITO-PVSK (20 mV s^{-1})-F	0.93	0.71	20.1	13.3
Glass/ITO-PVSK (200 mV s^{-1})-R	0.94	0.73	20.5	14.1
Glass/ITO-PVSK (200 mV s^{-1})-F	0.95	0.65	20.9	12.9
Glass/ITO-PVSK (2.4 V s^{-1}-R	0.94	0.73	20.8	14.3
Glass/ITO-PVSK (2.4 V s^{-1})-F	0.91	0.66	21.1	12.7
Flexible-PVSK (20 mV s^{-1})-R	0.90	0.80	19.4	14.0
Flexible-PVSK (20 mV s^{-1})-F	0.90	0.80	19.2	13.8
Flexible-PVSK (200 mV s^{-1})-R	0.91	0.80	19.4	14.1
Flexible-PVSK (200 mV s^{-1})-F	0.90	0.78	19.3	13.5
Flexible -PVSK (2.4 V s^{-1})-R	0.90	0.80	19.6	14.1
Flexible-PVSK (2.4 V s^{-1})-F	0.90	0.77	19.6	13.6

R: reverse scan direction, F: forward scan direction

Supplementary Table 3. Values for time-resolved PL characteristics of perovskite film on varied substrates.

Substrate	τ_1 (ns)	Ratio	τ_2 (ns)	Ratio
Glass/ITO/PEDOT:PSS	43.67	61.3%	165.95	38.7%
PET/Ag-mesh/PH1000/PEDOT:PSS	22.34	89.7%	129.40	10.3%

Supplementary Table 4. Values for time-resolved PL characteristics of perovskite film on PET/Ag-mesh/PH1000 substrate with different bending cycles at radius of 5 mm.

Bending condition	τ1 (ns)	Ratio	τ2 (ns)	Ratio
Origin	26.3	85.3%	117	14.7%
2000 bending cycles	25.1	86.2%	106	13.8%
5000 bending cycles	26.4	81.4%	104	18.6%

Supplementary Methods

Device characterization. The area of devices is 0.1 cm^2. *J–V* characteristics of photovoltaic cells were taken using a Keithley 2400 source measure unit under a simulated AM 1.5G spectrum. With an Oriel 9600 solar simulator, the light intensity was calibrated by KG-5 Si diode. The *J-V* measurements were carried out in nitrogen glove box. The devices were measured in both reverse scan (1.2V→0V) and forward scan (0V→1.2V) at different voltage scan-rates. The devices were taken out of nitrogen glove box for external quantum efficiencies measurement and EQE was measured by Enli tech (Taiwan) measurement system. For the bending test, we set up a simple tool to accommodate the flexible devices with one metal block on each side. One of them can be move back and forth along a railway with fixed starting and ending position according to the bending curvature. The moving of the block was manually controlled. For transient photovoltage and current measurements, a white light bias was generated from an array of diodes (Molex 180081-4320) to simulate 0.5 sun bias light working condition. A pulsed red dye laser (Rhodamine 6G, 590nm) pumped by a nitrogen laser (LSI VSL-337ND-S) was used as the perturbation source, with a pulse width of 4 ns and a repetition frequency of 10 Hz. The intensity of the perturbation laser pulse was controlled to maintain the amplitude of transient V_{OC} below 5 mV so that the perturbation assumption of excitation light holds. The voltages under open circuit and currents under short circuit conditions were measured over a 1 MΩ and a 50 Ω resistor, and were recorded on a digital oscilloscope (Tektronix DPO 4104B).

Structure and optical characterization. Film thicknesses were measured using a KLA Tencor profilometer. Transmittance spectra were acquired on a Cary 6000i UV-vis-NIR spectrophotometer. X-ray diffraction pattern (2θ scans) of perovskite deposited on the PET/Ag-mesh/PH1000/PEDOT:PSS substrates were obtained by using an X-ray diffractometer (Panalytical X'Pert Pro) under Cu-Kα radiation (λ=1.54050Å). The data were collected from 10 to 70° (2θ) with a 0.02° step size. The break region (17-28.3^{o}) in the XRD pattern (Fig. 5b and Supplementary Fig 8e) seeks

to remove the strong diffraction from the PET substrate. The top-view SEM images were collected by using an emission SEM (The Nova 230 NanoSEM), where the electron beam accelerated at in the range of 500 V to 30 kV. The cross-section SEM images of flexible pero-SCs were collected by using a Nova NanoLab™ DualBeam FIB- SEM/FIB, where the device was cut open by focused ion beam and subsequently collected the images of cross-section by field emission SEM. SKPM was performed on PET/Ag-mesh/PH1000 and PET/Ag-mesh/PH1000/PEDOT:PSS in air, and carried out on a Park XE-120 (Park Systems Corp. Suwon, Korea) atomic force microscopy using Cr/Au coated conducting tips (SC18, Mikromasch, Tallinn, Es tonia) with a resonance frequency of approximately 75 kHz and a spring constant of a pproximately 2.8 N m^{-1}. An AC voltage (3 V in amplitude and 10 kHz in frequency) is applied to the actuated cantilever, and the DC voltage applied to the tip that nullifie s the tip-sample interaction is collected as the SP signal. Through a feedback loop, this electrostatic force was nullified by a DC bias applied to the tip, while the DC bias served as a direct measurement of the surface potential. Steady-state photoluminescence (PL) was analyzed using Horiba Jobin Yvon system with an excitation at 640 nm. Time-resolved photoluminescence was obtained using the time correlated single-photon counting technique (Picoharp 300), and the excitation was provided by a picosecond diode laser at the wavelength of 768 nm with a repetition frequency of 1 MHz (PDL 800B).
